# An Energy-Efficient Gas–Oil Hybrid Servo Actuator with Single-Chamber Pressure Control for Biomimetic Quadruped Knee Joints

**DOI:** 10.3390/biomimetics11020131

**Published:** 2026-02-11

**Authors:** Mingzhu Yao, Zisen Hua, Huimin Qian

**Affiliations:** School of Artificial Intelligence, Anhui University of Science & Technology, Hefei 231131, China; 2023201663@aust.edu.cn (M.Y.); zcyyccg@163.com (H.Q.)

**Keywords:** hydraulic actuator, gas–oil hybrid, energy efficiency, quadruped robot, joint actuator

## Abstract

Legged robots inspired by animal locomotion require actuators with high power density, fast response, and robust force control, yet traditional valve-controlled hydraulic systems suffer from substantial energy losses and weak regeneration performance. Motivated by role allocation across gait phases in animal legs, where in-air positioning requires far less actuation effort than ground contact support and force modulation, this work proposes a novel gas–oil hybrid servo actuator, denoted GOhsa, for quadruped knee joints. GOhsa utilizes pre-charged high-pressure gas to pressurize hydraulic oil, converting the conventional dual-chamber pressure servo control into a single-chamber configuration while preserving the original piston stroke. This architecture enables bidirectional position–force control, enhances energy regeneration applicability, and improves operational efficiency. Theoretical modeling is conducted to analyze hydraulic stiffness and frequency-response characteristics, and a linearization-based force controller with dynamic compensation is developed to handle system nonlinearities. Experimental validation on a single-leg platform demonstrates significant energy-saving performance: under no-load conditions (simulating the swing phase), GOhsa achieves a maximum power reduction of 79.1%, with average reductions of 15.2% and 11.5% at inflation pressures of 3 MPa and 4 MPa, respectively. Under loaded conditions (simulating the stance phase), the maximum reduction reaches 28.0%, with average savings of 10.0% and 9.8%. Tracking accuracy is comparable to traditional actuators, with reduced maximum errors (13.7 mm/16.5 mm at 3 MPa; 15.0 mm/17.8 mm at 4 MPa) relative to the 16.6 mm and 18.1 mm errors of the conventional system, confirming improved motion stability under load. These results verify that GOhsa provides high control performance with markedly enhanced energy efficiency.

## 1. Introduction

Ground-based mobile robots are mainly classified into three categories: tracked robots, wheeled robots, and legged robots. Among these, legged robots exhibit superior mobility and terrain adaptability [1,2], with quadruped robots being particularly versatile across various environments, including search and rescue operations, electrical inspections, and geological exploration. Despite these advantages, energy efficiency remains a critical bottleneck that limits the widespread adoption of these robots, especially hydraulically driven systems [3,4]. Due to their high bandwidth, high stiffness, and excellent power-to-weight ratio [5], hydraulic actuators play a crucial role in high-load, high-mobility legged robots. To meet these growing demands, the development of more energy-efficient hydraulic servo systems remains an important research priority.

Over the past few decades, researchers have developed various strategies to improve the energy efficiency of legged robots. These approaches can be categorized into three main areas: motion optimization [6,7], passive energy storage mechanisms [8], and high-efficiency onboard hydraulic systems [9,10]. Among these methods, motion optimization has been the most commonly used and cost-effective approach. Examples include optimal pump speed control strategies based on model predictive control, or MPC, methods [11]; centroid wave gaits for hexapod robots that consider vertical centroid fluctuations [12]; and a kinodynamic MPC framework that integrates parallel elasticity and has achieved a 14.8% reduction in total energy consumption during hardware experiments [13]. However, the energy-saving potential of these methods depends heavily on the robot’s motion patterns, which constrains their overall effectiveness.

The second category, passive energy storage mechanisms, aims to store and release mechanical or hydraulic energy through compliant structures. Adding compliance elements to leg structures [14,15] or joint actuators [16,17] can achieve energy savings while reducing impact forces between robot feet and the ground, thereby improving dynamic stability and safety. Nevertheless, elastic elements can affect actuator bandwidth, and designing stiffness characteristics that match complex robot motion requirements remains challenging. In recent years, this concept has been extended to compact electro-hydrostatic actuators (EHAs) that support both active and regenerative energy functions. Prototype EHAs for wheel–legged robots have demonstrated rapid force response and notably higher transmission efficiency than conventional valve-controlled systems [18,19]. A recent review summarized efficient EHA architectures and bidirectional energy-recovery strategies [20]. These EHA-based systems integrate both active regeneration and passive energy storage mechanisms. However, their dual-chamber configurations still suffer from throttling and compression losses during low-load or return-stroke phases.

The third effective approach to energy saving focuses on improving individual hydraulic components or the entire hydraulic system [21,22]. Xue et al. [23] proposed a novel hydraulic cylinder with variable effective area. This design provides flexible and adjustable output force while enabling flow recovery through additional interior feeding chambers. Semini et al. [24] applied DC-DC switching buck converter theory to hydraulic system design, achieving approximately 75% energy savings. Xue et al. [22] proposed a multi-stage energy supplement system for quadruped robots based on varying drive requirements across different gait phases. Yang et al. [25] developed a three-chamber accumulator-based hydraulic energy saving system, achieving significant energy consumption reductions of up to 47.99% for hydraulic circuits. Liu et al. [26] proposed a novel controllable hydraulic accumulator, whose energy storage capacity is nearly 1.5 times greater than that of conventional designs. Yu et al. designed compact hydraulic power units, or HPUs, that adjust pump displacement and system pressure in real time to meet joint torque demands. This configuration reduces excess pump flow and decreases the overall system weight [27,28]. Compared with the previous two methods, this approach imposes fewer constraints on robot motion and provides a more direct means to improve energy efficiency. However, most existing systems still focus on the power supply side and are not specifically designed for the locomotion characteristics of legged robots. In addition, system complexity limits their practical application.

Building on these insights, and motivated by a widely observed role allocation in walking mammals, where swing-phase positioning requires far less actuation effort than stance-phase support and force modulation, this study introduces a novel gas–oil hybrid servo actuator, denoted GOhsa, specifically designed to address the unique kinematics and load characteristics of quadruped robot joints. To better accommodate the high-frequency, low-load piston retraction typical of legged locomotion, we redesign the traditional servo actuator’s annular chamber. The original piston retraction mechanism is replaced with a passive high-pressure gas chamber that drives return motion and provides impact buffering. The actuator’s integrated and lightweight design minimizes added mass and volume, while the deliberate reduction of hydraulic stiffness provides inherent buffering capacity. Based on theoretical mathematical modeling, the hydraulic stiffness characteristics and driving response capabilities were analyzed to verify bidirectional controllability for small loads. To ensure system stability in force control strategies for quadruped robots, a force controller for GOhsa is designed using linearization theory. The controller’s stability, along with the actuation and energy-saving capabilities of GOhsa, was verified by conducting tests on a single-leg evaluation setup.

The remainder of this paper is organized as follows: Section 2 presents the detailed structure of GOhsa and analyzes its hydraulic stiffness and response characteristics. Section 3 describes the design procedure for the GOhsa force controller. Section 4 presents experimental validation results. Finally, Section 5 provides conclusions. The main contributions of this work are summarized as follows:

(1) Motivated by role allocation across gait phases in mammalian leg actuation, where in-air positioning demands much smaller actuation effort than ground contact support and force modulation, we propose a gas–oil hybrid servo actuator, denoted GOhsa. A precharged gas–oil module passively pressurizes the ring chamber, simplifying conventional dual chamber pressure servo control to active regulation of a single chamber while preserving piston stroke and bidirectional position and force control.
(2) A mathematical model is developed to characterize the hydraulic stiffness and frequency response of GOhsa and to analyze how gas precharge pressure affects actuation dynamics.
(3) A force controller based on linearization with dynamic compensation is designed and validated experimentally on a single leg platform, achieving tracking performance comparable to that of a conventional actuator while reducing power demand during piston retraction.

## 2. Gas–Oil Hybrid Drive Servo Actuator for Quadruped Robot SCalf-II

This section first describes the biomimetic inspiration and design rationale, as well as the quadruped robot leg structure and its corresponding joint actuators, then presents the design principle of the proposed gas–oil hybrid servo actuator (GOhsa), and analyzes the hydraulic stiffness characteristics and frequency response of the system.

### 2.1. Biomimetic Inspiration and Design Rationale

Motivated by the morphology and actuation traits of walking mammals, this work translates a functional principle that depends on gait phase into an actuator architecture. In cursorial mammals such as canines, distal joint kinematics during steady gait are often described by sagittal plane flexion and extension and can be repeatable within a uniform breed [29]. Muscles spanning these joints typically provide modest tensile effort for in-air leg positioning, whereas ground contact demands much larger forces for body weight support and active force modulation. These tasks must still be executed at comparable actuation speeds. This separation between low force positioning and high force support across gait phases motivates our architecture choice in GOhsa. We reserve single-chamber active pressure regulation for the high-load support requirement and simplify the return pathway by passively pressurizing the ring chamber, improving energy efficiency without sacrificing bidirectional force and position controllability.

### 2.2. Conceptual Design of GOhsa

**Figure 1:**
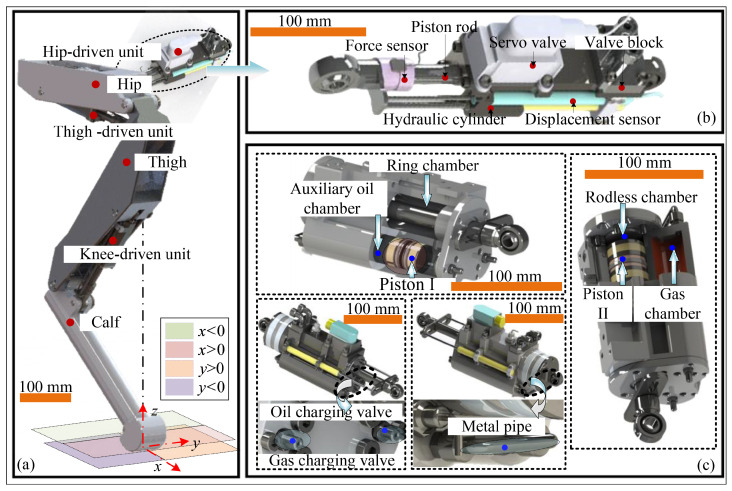
Robot leg and joint-driven actuator. (**a**) Structure of the leg. (**b**) Traditional joint-driven unit. (**c**) GOhsa.

Figure 1 shows the conventional leg structure of hydraulically driven quadruped robots (Figure 1a) and their joint actuators (Figure 1b). The traditional leg structure consists of four main components: hip, thigh, calf, and joint actuator. Each leg has three degrees of freedom, or DOFs, ensuring a wide range of motion for the robot. Figure 1b shows the joint actuator configuration commonly used in hydraulically driven quadruped robots. It consists of a hydraulic cylinder, displacement sensor, force sensor, valve block, and servo valve. The highly integrated design significantly reduces external hydraulic hoses and effectively improves the actuator’s dynamic characteristics.

Hydraulically driven quadruped robots exhibit a comparable division of actuation roles between swing and support, consistent with the gait-phase-dependent role allocation described for walking mammals in Section 2.1. To expand the feasible foothold search range for the robot torso under unstable conditions while meeting height adjustment requirements during stationary and locomotion phases, the foot workspace is typically designed with approximate symmetry in both lateral and sagittal planes, using the vertical line through the thigh pitch joint as the boundary. Consequently, during the support phase, ground reaction forces cause alternating load directions on the hip yaw joint and thigh pitch joint. However, the calf pitch joint experiences consistent compression from ground reaction forces throughout this phase. During the swing phase, without environmental reaction forces, all three joint actuators only need to overcome gravitational loads and inertial forces of the linked segments. As the terminal actuator in the kinematic chain, the calf pitch joint experiences the smallest load forces among the three joints, despite being influenced by coupled motions from upstream linkages.

Motivated by this swing and support contrast, the key challenge in energy-efficient joint actuator design is meeting the actual robot motion requirements without altering the original design specifications. For legged robots, achieving torso stability under normal conditions while ensuring rapid and accurate foot placement during unstable conditions requires stringent position servo accuracy for the foot and force servo precision for the joints. Although advanced control algorithms have significantly improved position and force servo accuracy, enhanced pressure differential regulation does not fundamentally address the issue of high energy consumption in joint actuators.

For hydraulic transmission systems, pressure and flow rate jointly determine the operational energy consumption. To meet high mobility requirements while balancing control complexity, onboard hydraulic servo systems in quadruped robots typically use constant-pressure variable-displacement architecture, where system pressure is determined by the maximum load capacity. However, this architecture causes excessive throttling power loss during low-load phases. Disregarding factors such as hydraulic oil compressibility, system pressure losses, and leakage, the required system flow rate is approximately equal to the hydraulic cylinder piston operating velocity. Therefore, without altering maximum system driving capacity, the most feasible method to reduce energy consumption is decreasing the supplied flow rate. However, this strategy is impractical for hip and thigh actuators, which experience highly irregular loads and motion patterns. In contrast, it is applicable to the calf actuator, which experiences relatively small loads during swing phases and primarily sustains compressive forces during support phases.

Based on this analysis, two straightforward approaches could theoretically improve energy efficiency without altering the existing quadruped robot configuration or affecting motion properties. The first approach uses accumulator-based energy recovery systems commonly found in construction machinery. However, the energy-saving effectiveness of these systems depends entirely on load type and hydraulic-cylinder piston-motion direction. When the driving force is insufficient, the main oil source must still intervene to regulate pressure and flow. Therefore, this approach is clearly unsuitable for quadruped robots with highly variable motion patterns. The second approach uses a single-acting hydraulic cylinder driven by an auxiliary mechanical spring. However, the added spring reduces the effective piston stroke and may impair the smoothness of piston movement due to friction between the spring, piston rod, and cylinder barrel. Furthermore, achieving stiffness characteristics that align with the motion requirements presents a significant challenge. Considering these factors, Figure 1c shows the conceptual design of the joint actuator proposed in this study. In this figure, Piston I refers to the sliding piston in the auxiliary oil chamber, and Piston II refers to the main piston separating the rodless and rod chambers.

Compared to traditional dual-chamber actuators, GOhsa introduces a passive high-pressure gas chamber that is integrated into the cylinder barrel. This chamber connects via external metal piping to an auxiliary oil chamber symmetrically positioned on the opposite side. A sliding piston divides the auxiliary oil chamber into two sections: one side connects to the high-pressure gas chamber to serve as a gas flow channel, while the other side functions as a hydraulic oil passage. The oil side of the auxiliary chamber communicates with the ring side of Piston II through an internal bore. The high-pressure gas chamber, auxiliary oil chamber, and the ring chamber collectively form a sealed variable-volume space. The gas pressure in the gas chamber primarily determines the hydraulic oil pressure within the auxiliary chamber. The sliding piston isolates the auxiliary oil chamber, providing functionality similar to a piston-type accumulator. Adjustable check valves integrated into the end covers enable flexible gas and hydraulic oil charging and discharging. With this configuration, the GOhsa servo valve no longer needs to regulate pressure in the ring chamber. Instead, precise force and position control can be achieved by managing only the flow rate and pressure of the rodless chamber. This configuration enables GOhsa to offer advantages of compactness, high energy efficiency, and sufficient response speed. Compared with variable working area hydraulic actuators and the Buck converter hydraulic drive schematic, the proposed structure improves actuation energy efficiency with fewer electro-hydraulic servo elements and reduced serial piping, while using a single electro-hydraulic servo valve also greatly reduces the signal measurement and logic control cost of complex control loops. From the perspective of energy saving effectiveness, because the proposed structure completely eliminates the need to regulate the rod chamber oil pressure and flow, its energy saving advantage is evident compared with compliant variable stiffness hydraulic actuators that only buffer energy during overpressure events. Therefore, for quadruped robot knee joints that frequently undergo low-load retraction and return strokes, GOhsa is particularly well suited.

### 2.3. General Problem Formulation and Performance Analysis

#### 2.3.1. Hydraulic Stiffness $K_{a}$

**Figure 2:**
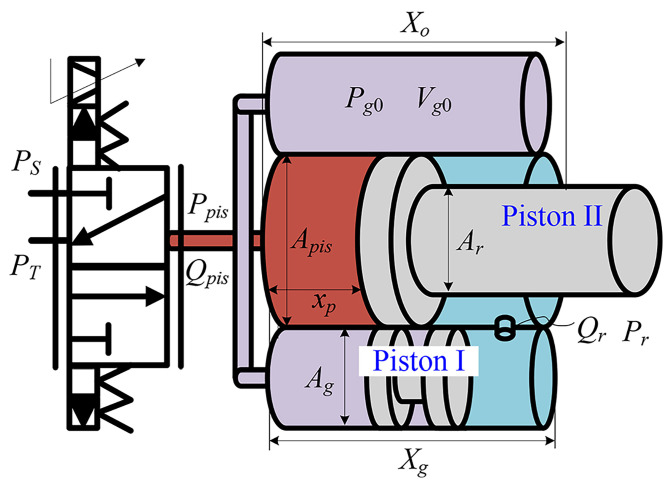
Sketch map of GOhsa.

As shown in Figure 2, the hydraulic actuator output force $f_{h}$ is determined by the force balance between the rodless and ring chambers. In the figure, $A_{pis}$ and $A_{r}$ denote the effective piston areas of the rodless and rod sides, respectively, while $P_{pis}$ and $P_{r}$ represent the corresponding chamber pressures. The variables $Q_{pis}$ and $Q_{r}$ are the volumetric flow rates entering the rodless and ring chambers, and $x_{p}$ denotes the displacement of Piston II (the main piston), with positive $x_{p}$ increasing the rodless chamber volume. $A_{g}$ is the gas-side effective area of Piston I; $P_{g0}$ and $V_{g0}$ are the initial gas pressure and gas volume, respectively; $X_{o}$ is the total stroke of Piston II; and $X_{g}$ is the total stroke of the gas and auxiliary oil chambers. $P_{S}$ and $P_{T}$ refer to the supply and return pressures of the hydraulic circuit. For the static stiffness analysis, external and internal leakage as well as frictional damping are neglected, and the force dynamics can be expressed as:

(1) $${\dot{f}}_{h}=A_{pis}{\dot{P}}_{pis}-A_{r}{\dot{P}}_{r}$$

The relationship between the two effective piston areas can be written as $A_{r}=\alpha A_{pis}$, where α denotes the area ratio between the two sides of the piston.

According to the continuity equation, the pressure dynamics of $P_{pis}$ can be expressed as [30]:

(2) $${\dot{P}}_{pis}=\frac{\beta_{e}\left(-A_{pis}{\dot{x}}_{p}+Q_{pis}\right)}{V_{pis}}\;$$

where $\beta_{e}$ represents the effective bulk modulus of the hydraulic oil, and $V_{pis}$ is the total volume of the rodless chamber, including the pipe volume $V_{pl}$.

The initial gas chamber volume is determined by the structural volume of the gas chamber and auxiliary oil chamber, the design dead volume of the auxiliary oil chamber, and the stroke of the main piston. This represents the total gas chamber volume when the main piston is fully retracted. ${\dot{P}}_{r}$ can be expressed according to Boyle’s law:

(3) $${\dot{P}}_{r}=\frac{A_{r}V_{g0}P_{g0}{\dot{x}}_{p}}{{\left(V_{g0}-A_{r}x_{p}\right)}^{2}}\;$$

where $V_{g0}$ can be expressed as $\left(2A_{g}X_{g}-V_{dead}\right)$, with $V_{dead}$ denoting the designed dead volume. Under the assumption of an impermeable and rigidly coupled separating piston, the ring chamber oil pressure is approximately equal to the gas pressure; hence, the gas-side pressure rate ${\dot{P}}_{g}$ can be expressed in the same form as Equation (3).

Considering Equations (1) through (3), the force dynamics of GOhsa can be expressed as:

(4) $${\dot{f}}_{h}=\frac{A_{pis}\beta_{e}\left(Q_{pis}-A_{pis}{\dot{x}}_{p}\right)}{V_{pis}}-\frac{A_{r}^{2}V_{g0}P_{g0}{\dot{x}}_{p}}{{\left(V_{g0}-A_{r}x_{p}\right)}^{2}}\;$$

Each working chamber of GOhsa can then be characterized by a transmission stiffness:

(5) $$\left\{\begin{array}{ccc} K_{pis} & = & \frac{A_{pis}\beta_{e}}{V_{pis0}+A_{pis}x_{p}}\;\\ K_{r} & = & \frac{A_{r}V_{g0}P_{g0}}{{\left(V_{g0}-A_{r}x_{p}-V_{pis0}\right)}^{2}}\; \end{array}\right.$$

where $V_{pis0}$ denotes the initial volume of the rodless chamber.

Similarly, the hydraulic stiffness of the actuator can be defined as:

(6) $$K_{a}=K_{pis}+K_{r}=\frac{A_{pis}\beta_{e}}{V_{pis0}+A_{pis}x_{p}}+\frac{A_{r}V_{g0}P_{g0}}{{\left(V_{g0}-A_{r}x_{p}-V_{pis0}\right)}^{2}}\;$$

Hydraulic stiffness $K_{a}$ serves as a critical performance metric for evaluating the dynamic response, control precision, and operational reliability of hydraulic systems. The relationship between $K_{a}$ and $x_{p}$ is nonlinear, and its variation is also affected by $P_{g0}$ and $V_{g0}$. By differentiating Equation (6) with respect to $V_{g0}$ and $P_{g0}$, respectively, we obtain:

(7) $$\left\{\begin{array}{ccc} \frac{\partial K_{a}}{\partial V_{g0}} & = & -\frac{A_{r}P_{g0}\left(V_{g0}+A_{r}x_{p}+V_{pis0}\right)}{{\left(V_{g0}-A_{r}x_{p}-V_{pis0}\right)}^{3}}\;\\ \frac{\partial K_{a}}{\partial P_{g0}} & = & \frac{A_{r}V_{g0}}{{\left(V_{g0}-A_{r}x_{p}-V_{pis0}\right)}^{2}}\; \end{array}\right.$$

To ensure full piston stroke, the gas chamber volume should not reach zero when the main piston is fully retracted, indicating that $(A_{g}X_{g}-A_{r}X_{o})>0$ must always hold. Thus, $K_{a}$ decreases with increasing $V_{g0}$, and the rate of decrease gradually slows down. In contrast, $K_{a}$ increases with increasing $P_{g0}$, and this rate accelerates as $x_{p}$ increases. For system safety, the maximum gas pressure should be less than 1.5 times the initial gas pressure, which indicates that $V_{g0}$ must exceed 1.5$A_{r}X_{o}$ according to Boyle’s law. However, an oversized gas chamber negatively affects the integrated design of the actuator, thereby reducing its adaptability to robot leg design.

**Figure 3:**
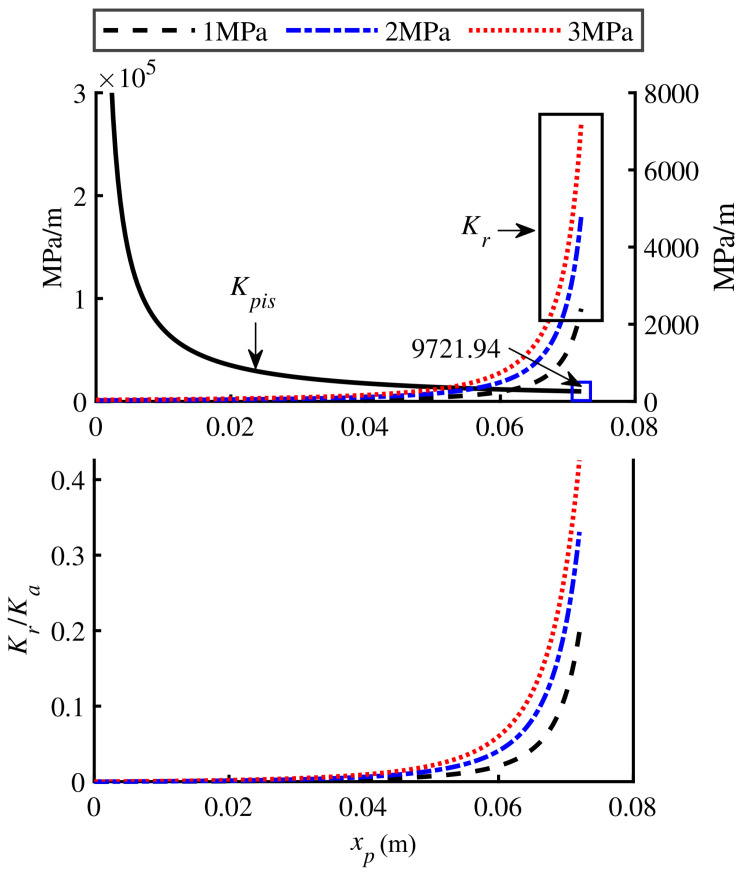
Hydraulic stiffness characteristics of GOhsa.

Based on the dimensions of joint-driven units used by quadruped robot SCalf-II (as listed in Table 1) while ensuring full compatibility with the original structure, the length of the gas chamber and the auxiliary oil chamber should not exceed 0.2 m (the geometric length of the cylinder barrel). Assuming a preliminary inner diameter of 0.02 m for both chambers, the theoretical minimum lengths of both chambers are calculated to be 0.065 m. Further accounting for the length of the sliding piston and the influence of the safety dead zone on the effective stroke, the effective lengths of both chambers are determined to be 0.15 m. Based on the above parameters, the variations of $K_{pis}$ and $K_{r}$ with respect to $x_{p}$ are shown in Figure 3.

The results show that $K_{pis}$ and $K_{r}$ exhibit opposite nonlinear trends as $x_{p}$ increases. As the pre-inflation pressure increases, $K_{r}$ also increases significantly, but the magnitude of its variation is notably smaller than that of $K_{pis}$. Within the examined range, the contribution of $K_{r}$ to $K_{a}$ does not exceed 45%.

#### 2.3.2. Response Capability

This section further discusses the response capability of GOhsa by considering internal friction, damping, and the dynamic characteristics of the pistons. The dynamics of the main piston and sliding piston can be described as follows:

(8) $$\left\{\begin{array}{ccc} m_{s}{\ddot{x}}_{s} & = & A_{g}\left(P_{pis}-P_{r}\right)-f_{s}\left({\dot{x}}_{s}\right)\;\\ m_{p}{\ddot{x}}_{p} & = & A_{pis}P_{pis}-A_{r}P_{r}-f_{p}\left({\dot{x}}_{p}\right)\; \end{array}\right.$$

where $m_{s}$ and $m_{p}$ are the masses of the sliding and main pistons, respectively, and $x_{s}$ denotes the displacement of the sliding piston. The steady-state friction model is adopted, with $f_{s}$ and $f_{p}$ representing the friction forces acting on the sliding and main pistons, respectively. It combines static, Coulomb, and viscous effects according to the classical Stribeck friction law [31,32] and is expressed as:

(9) $$f\left(\dot{x}\right)=\left[F_{C}+\left(F_{S}-F_{C}\right)e^{-{\left(\dot{x}/{\dot{X}}_{S}\right)}^{\beta }}\right]\mathrm{sign}\left(\dot{x}\right)+B\dot{x}$$

where *x* denotes displacement, $F_{C}$ and $F_{S}$ represent the Coulomb and static friction forces, ${\dot{X}}_{S}$ and β denote the velocity scale and exponent of the Stribeck effect, and *B* is the viscous damping coefficient.

Neglecting internal and external leakage, the gas chamber pressure dynamics ${\dot{P}}_{g}$ can still be described by Equation (3), whereas the ring chamber pressure considering oil compressibility can be described as:

(10) $${\dot{P}}_{r}=\frac{\beta_{e}\left(-\alpha A_{pis}{\dot{x}}_{p}+A_{g}{\dot{x}}_{s}\right)}{A_{r}\left(X_{o}-x_{p}\right)+A_{g}\left(X_{g}-x_{s}\right)}$$

Furthermore, the controlled flow into the rodless chamber $Q_{pis}$ follows the classical orifice equation of a directional control valve [33,34]. The model is further refined by incorporating valve current–displacement proportionality and flow-direction switching through the sign-dependent function $\Psi (x_{v})$, as follows:

(11) $$Q_{pis}=\Psi \left(x_{v}\right)K_{v}\frac{x_{v}^{max}}{i_{v}^{max}}i_{v}\sqrt{P_{S}-P_{pis}}-\Psi \left(-x_{v}\right)K_{v}\frac{x_{v}^{max}}{i_{v}^{max}}i_{v}\sqrt{P_{pis}-P_{T}}$$

where $x_{v}^{max}$ and $i_{v}^{max}$ represent the maximum valve spool stroke and maximum control current, respectively; $i_{v}$ and $x_{v}$ are the control current and corresponding valve spool displacement; $K_{v}$ represents the valve gain. The function $\Psi (x_{v})$ is defined as:

(12) $$\Psi \left(x_{v}\right)=\left\{\begin{array}{cc} 1, & x_{v}\geq 0\\ 0, & x_{v}<0 \end{array}\right.$$

The relationship between the valve spool position $x_{v}$ and the control current $i_{v}$ is modeled as a second-order system:

(13) $$x_{v}=\frac{K_{1}i_{v}}{s^{2}+2\omega_{v}\xi_{v}s+\omega_{v}^{2}}=G_{v}K_{1}i_{v}$$

where $K_{1}$ is the current gain; *s* is the Laplace variable; $\omega_{v}$ and $\xi_{v}$ are the valve spool’s natural frequency and damping ratio, respectively; and $G_{v}$ denotes the second-order spool dynamics.

Taking into account Equations (2), (3), and (8)–(13), a simulation model is established in the Simulink environment. Based on empirical data from conventional hydraulic cylinders, the key parameters involved in the model are listed in Table 1 and Table 2. The oil supply pressure $P_{S}$ and returning oil pressure $P_{T}$ are set to 20 MPa and 0 MPa, respectively. The flow rate of the electric-hydraulic servo valve is 28 L/min.

**Figure 4:**
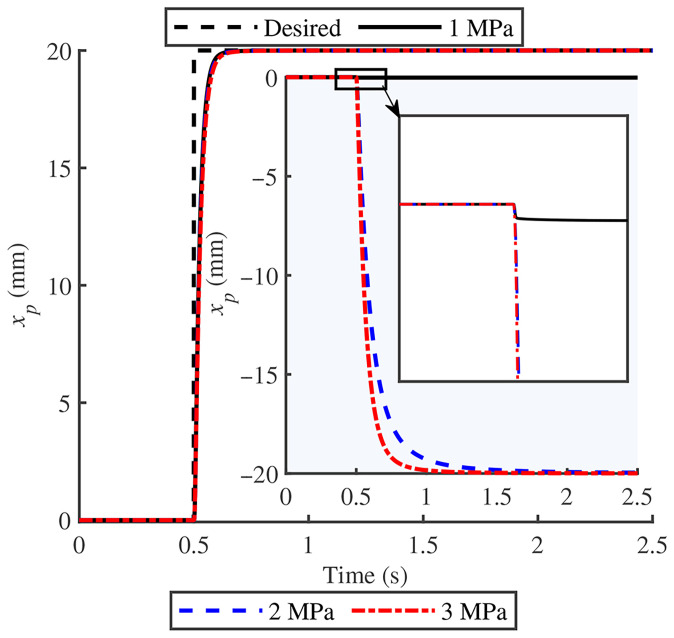
Step response of the main piston displacement $x_{p}$ under no-load excitation; the main plot shows extension and the inset shows retraction.

**Figure 5:**
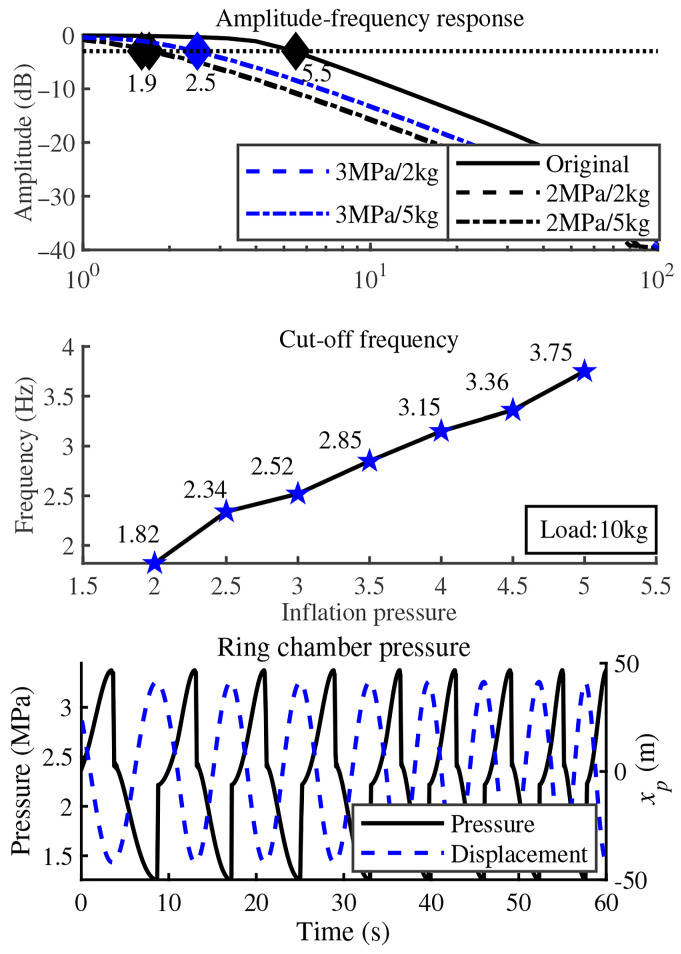
Frequency-domain characteristics and chamber pressure under sinusoidal sweep excitation.

Figure 4 presents the displacement response curve of GOhsa under step signal excitation with no load. The results show that changing the initial gas chamber inflation pressure does not affect the piston extension stroke, while the retraction stroke response speed shows a positive correlation with initial inflation pressure. When the pre-inflation pressure is set at 1 MPa, the desired motion does not exhibit an immediate response. This phenomenon confirms the existence of a minimum starting pressure in practical hydraulic actuators. Figure 5 presents the sinusoidal sweep response results under different initial inflation pressures and loads, with signal amplitude set to 0.035 m. Compared with traditional joint actuators, the bandwidth response of GOhsa decreases markedly within the investigated range, with no noticeable improvement under minor load variations. When the load remains constant, GOhsa cut-off frequency increases with increasing initial inflation pressure, which is consistent with basic physical principles. Given that quadruped robot gait speeds are relatively low, the slight decrease in GOhsa response bandwidth does not substantially impact the robot’s motion capability.

Figure 5 also simultaneously presents the real-time tracking characteristics between the ring chamber pressure and piston displacement of the actuator, showing only the low-speed operation region. Results reveal that during piston directional reversal, the ring chamber pressure maintains observable step-change characteristics, while exhibiting limited overall pressure oscillations with relative lower initial inflation pressure (2 MPa). Notably, the displacement response also maintains its amplitude without appreciable decay even at low excitation frequencies. Considering these results and the step response curve, when the gas chamber pre-inflation pressure exceeds a certain threshold, the ring chamber pressure response can theoretically meet the required operational specifications. The 3 MPa/2 kg and 3 MPa/5 kg curves are nearly identical in the frequency domain, so their blue dashed lines and cut-off markers have a high degree of overlap.

## 3. Controller Design

According to the analysis above, a significant difference between traditional joint actuators and GOhsa is that the ring chamber pressure can be calculated from the sliding piston displacement, indicating that $P_{r}$ becomes an uncontrollable parameter once the desired piston position has been reached. Therefore, the control model derivation is based on the assumption of low-load operation, where larger external forces are generated only as compression loads. This is consistent with the original design intention of this structure.

Considering that the servo valve natural frequency $\omega_{v}$ is several times faster than the pressure and loading dynamics of the quadruped robot, Equation (12) can be simplified by assuming $G_{v}$ = 1:

(14) $$\Delta x_{v}=K_{1}\Delta i_{v}\;$$

Considering Equations (11) through (14), the rodless chamber flow rate $Q_{pis}$ can be expressed as:

(15) $$Q_{pis}=\left\{\begin{array}{c} K_{v}K_{1}i_{v}\sqrt{P_{S}-P_{pis}}\;x_{v}\geq 0\;\\ K_{v}K_{1}i_{v}\sqrt{P_{pis}-P_{T}}\;x_{v}<0\; \end{array}\right.$$

Given the high nonlinearity of the mechatronic-hydraulic system in the servo valve, obtaining control laws based on precise models becomes difficult. Therefore, the nonlinear model represented by Equation (11) can be linearized around the equilibrium point $P_{0}$ = ($P_{pis0}$,$i_{v0}$) [35], where $P_{pis0}$ is the equilibrium pressure in the rodless chamber and $i_{v0}$ is the corresponding valve control current at the equilibrium point. In this work, the point ($P_{T}$,0) is chosen as the equilibrium point. Expanding Equation (15) using Taylor series expansion around the equilibrium point $P_{0}$ yields the following expression:

(16) $$\Delta Q_{pis}=K_{Q}^{pis}\Delta I_{v}\;$$

where $\Delta I_{v}$ denotes the incremental valve control current obtained by linearizing the original current $i_{v}$ around its equilibrium point. For simplicity, $I_{v}$ is used in the linearized model to represent the valve control input.

Where

(17) $$K_{Q}^{pis}=\frac{\partial Q_{pis}}{\partial I_{v}}{|}_{P_{0}}=\left\{\begin{array}{c} K_{v}K_{1}\sqrt{P_{S}-P_{pis0}}\;x_{v}\geq 0\;\\ K_{v}K_{1}\sqrt{P_{pis0}-P_{T}}\;x_{v}<0\; \end{array}\right.$$

From the analysis in the previous section, once the pre-inflation pressure exceeds a certain threshold, the dynamic characteristics of Piston I do not significantly affect the overall actuator output performance. Therefore, only the force characteristics of Piston II are considered. Neglecting damping and Coulomb friction initially, the dynamics of the output force $f_{h}$ can be obtained by taking the time derivative:

(18) $${\dot{f}}_{h}=\beta_{e}A_{pis}\frac{K_{Q}^{pis}\Delta I_{v}}{V_{pis}}-{\dot{x}}_{p}\left[\frac{\beta_{e}A_{pis}^{2}}{V_{pis}}+\frac{A_{r}^{2}V_{g0}P_{g0}}{{\left(V_{g0}-A_{r}x_{p}\right)}^{2}}\right]\;$$

Assuming $V_{pis}$ and $x_{p}$ are constant (denoted as $V_{pis0}$ and $x_{p0}$), the above equation can be linearized as follows:

(19) $${\dot{f}}_{h}=K_{I}\Delta I_{v}-K_{\dot{X}}\Delta {\dot{x}}_{p}\;$$

where

(20) $$\left\{\begin{array}{c} K_{I}=\frac{\beta_{e}A_{pis}K_{Q}^{pis}}{V_{pis0}}\;\\ K_{\dot{X}}=\frac{\beta_{e}A_{pis}^{2}}{V_{pis0}}+\frac{A_{r}^{2}V_{g0}P_{g0}}{{\left(V_{g0}-A_{r}x_{p0}\right)}^{2}}\; \end{array}\right.$$

By further considering viscous friction effects, the actuator output force can be expressed as follows:

(21) $$\Delta f=\Delta f_{h}^{d}+\Delta f_{e}=\Delta f_{h}-B\Delta {\dot{x}}_{p}+\Delta f_{e}\;$$

where $\Delta f_{h}^{d}$ denotes the net hydraulic driving force (excluding viscous friction), and $f_{e}$ denotes the external load force.

According to Newton’s law, the following relation should be satisfied:

(22) $$\Delta {\ddot{x}}_{p}=\frac{\Delta f_{h}^{d}}{m}+\frac{\Delta f_{e}}{m}\;$$

where *m* denotes the equivalent moving mass, which includes Piston II and the reflected load.

Based on the analysis above, the state-space representation of the actuator model can be expressed by combining Equations (19) through (22):

(23) $$\left[\begin{array}{c} \Delta {\dot{x}}_{p}\;\\ \Delta {\ddot{x}}_{p}\;\\ \Delta {\dot{f}}_{h}\; \end{array}\right]=\left[\begin{array}{ccc} 0 & 1 & 0\;\\ 0 & -\frac{B}{m} & \frac{1}{m}\;\\ 0 & -K_{\dot{X}} & 0\; \end{array}\right]\left[\begin{array}{c} \Delta x_{p}\;\\ \Delta {\dot{x}}_{p}\;\\ \Delta f_{h}\; \end{array}\right]+\left[\begin{array}{cc} 0 & 0\\ 0 & \frac{1}{m}\;\\ K_{I} & 0\; \end{array}\right]\left[\begin{array}{c} \Delta I_{v}\;\\ \Delta f_{e}\; \end{array}\right]$$

(24) $$\left[\begin{array}{c} \Delta x_{p}\;\\ \Delta f_{h}^{d}\; \end{array}\right]=\left[\begin{array}{ccc} 1 & 0 & 0\;\\ 0 & -B & 1\; \end{array}\right]\left[\begin{array}{c} \Delta x_{p}\;\\ \Delta {\dot{x}}_{p}\;\\ \Delta f_{h}\; \end{array}\right]$$

Based on the equations above, the transfer functions between input and output variables can be derived as follows:

(25) $$\Delta x_{p}=\frac{K_{I}}{s(ms^{2}+Bs+K_{\dot{X}})}\Delta I_{v}+\frac{1}{ms^{2}+Bs+K_{\dot{X}}}\Delta f_{e}\;$$

(26) $$\Delta f_{h}^{d}=\frac{K_{I}ms^{2}}{s(ms^{2}+Bs+K_{\dot{X}})}\Delta I_{v}-\frac{K_{\dot{X}}+Bs}{ms^{2}+Bs+K_{\dot{X}}}\Delta f_{e}\;$$

Using Equations (25) and (26) to eliminate $f_{e}$, $f_{h}^{d}$ can be further expressed as:

(27) $$\Delta f_{h}^{d}=\frac{K_{I}\Delta I_{v}-\left(Bs^{2}+K_{\dot{X}}s\right)\Delta x_{p}}{s}\;$$

Assume that

(28) $$\Delta I_{v}=\Delta I_{c}+\Delta I_{x}\;$$

and define

(29) $$\Delta I_{x}=\frac{\left(Bs^{2}+K_{\dot{X}}s\right)\Delta x_{p}}{K_{I}}=K_{x1}\Delta {\ddot{x}}_{p}+K_{x2}\Delta {\dot{x}}_{p}\;$$

Therefore, Equation (28) can be expressed as:

(30) $$\Delta f_{h}^{d}=\frac{K_{I}\left(\Delta I_{c}+\Delta I_{x}\right)-\left(Bs^{2}+K_{\dot{X}}s\right)\Delta x_{p}}{s}=\frac{K_{I}}{s}\Delta I_{c}=G\left(s\right)\Delta I_{c}\;$$

Clearly, G(s) alone cannot ensure system stability. Therefore, a feedback controller must be introduced into the system. In this study, a proportional integral derivative controller, abbreviated PID, with its simplicity and robustness [36], is chosen as the first option. To reduce force sensor noise and avoid numerical differentiation errors, the control law of $I_{c}$ can be expressed as:

(31) $$\Delta I_{c}=k_{p}\left(\Delta f_{h}^{des}-\Delta f_{h}^{fed}\right)+k_{i}\int \left(\Delta f_{h}^{des}-\Delta f_{h}^{fed}\right)dt\;$$

where $k_{p}$ and $k_{i}$ are the proportional and integral gains of the controller; $f_{h}^{des}$ and $f_{h}^{fed}$ represent the desired and feedback values of the load force, respectively.

The closed-loop transfer function between $f_{h}^{fed}$ and $f_{h}^{des}$ can then be derived as:

(32) $$\frac{f_{h}^{fed}}{f_{h}^{des}}=\frac{K_{I}k_{p}s+K_{I}k_{i}}{s^{2}+K_{I}k_{p}s+K_{I}k_{i}}\;$$

Solving the characteristic polynomial, the eigenvalues are:

(33) $$s_{1,2}=\frac{-K_{I}k_{p}\pm \sqrt{K_{I}^{2}k_{p}^{2}-4K_{I}k_{i}}}{2}\;$$

From the above relations, the following conclusions can be drawn:

(1) As long as $k_{p}$ and $k_{i}$ are positive, when $K_{I}^{2}k_{p}^{2}-4K_{I}k_{i}>=0$, $s_{1}$ and $s_{2}$ remain in the left half-plane;
(2) If $K_{I}^{2}k_{p}^{2}-4K_{I}k_{i}<0$, $s_{1}$ and $s_{2}$ move to the left when $k_{p}$ is increased;
(3) $\sqrt{K_{I}k_{i}}$ represents the system natural frequency. Higher values of $\sqrt{K_{I}k_{i}}$ result in faster response rate but increase the vibration risk. Thus, $k_{i}$ should be set within a certain range.

## 4. Simulation and Experiment

This section further verifies the practicality and dynamic output response characteristics of GOhsa on the quadruped robot, both in simulation and on the physical platform.

### 4.1. Simulation and Analysis

To validate the practical performance of the actuator proposed in this study on the quadruped robot platform, a full-scale dynamic model of the SCalf-II quadruped robot was developed using the Simcenter Amesim (2020.1) multi-physics coupling simulation environment [37]. Since the GOhsa is only used for the lower leg joint drive, a two-dimensional simplified modeling approach was employed and validated using a typical trot gait under position control. The structural parameters of the SCalf-II robot, along with the derivation of its leg kinematic and dynamic models, are detailed in reference [38], while the additional parameters for the GOhsa components are provided in Table 3, with the performance parameters of the servo valve configured based on the actual hardware setup. The rest parameters can be found in Table 1.

**Figure 6:**
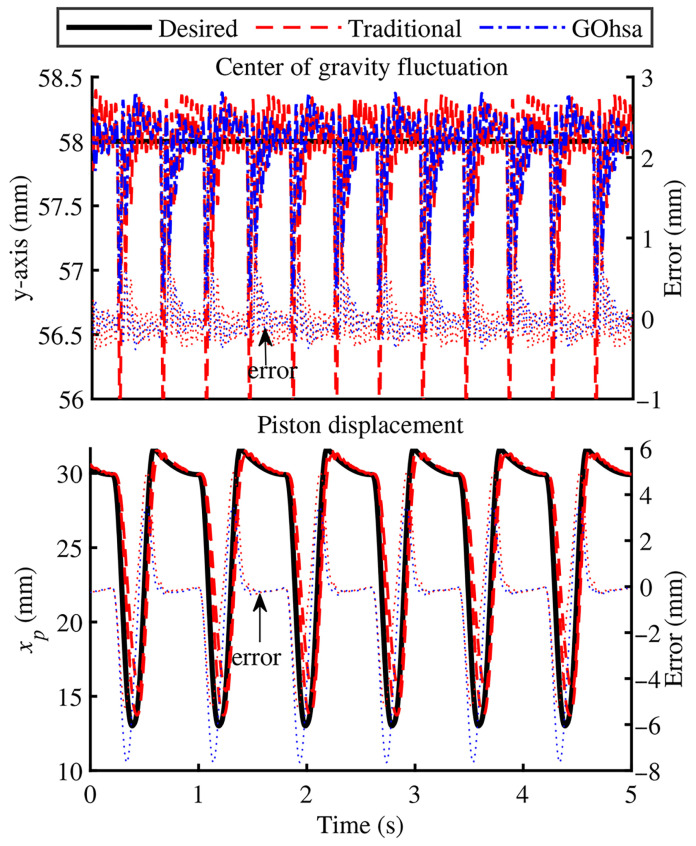
Body fluctuation and piston displacement.

In the simulation process, the robot’s torso mass was set to 120 kg, with a standing height maintained at 580 mm. The robot followed a planned trajectory with a step frequency of 1.25 Hz, a step length of 200 mm, and a leg lift height of 100 mm for periodic movement. The initial inflation pressure of GOhsa was set to 4 MPa. Figure 6 illustrates the vertical fluctuation of the center of gravity of the robot’s torso, as well as the real-time displacement tracking curve of the knee-driven unit in the left front leg. To facilitate comparison, the tracking error between the actual output and the desired trajectory is overlaid in each sub-figure. The experimental results indicate that, under the same parameter configuration, the GOhsa experimental group exhibits smoother vertical torso fluctuations during the foot–ground contact phase compared to the traditional servo actuator group. However, no significant difference in trajectory tracking errors is observed between the two groups. Further analysis of the actuator displacement curve reveals that the GOhsa has achieved dynamic tracking accuracy comparable to that of the traditional servo actuator.

**Figure 7:**
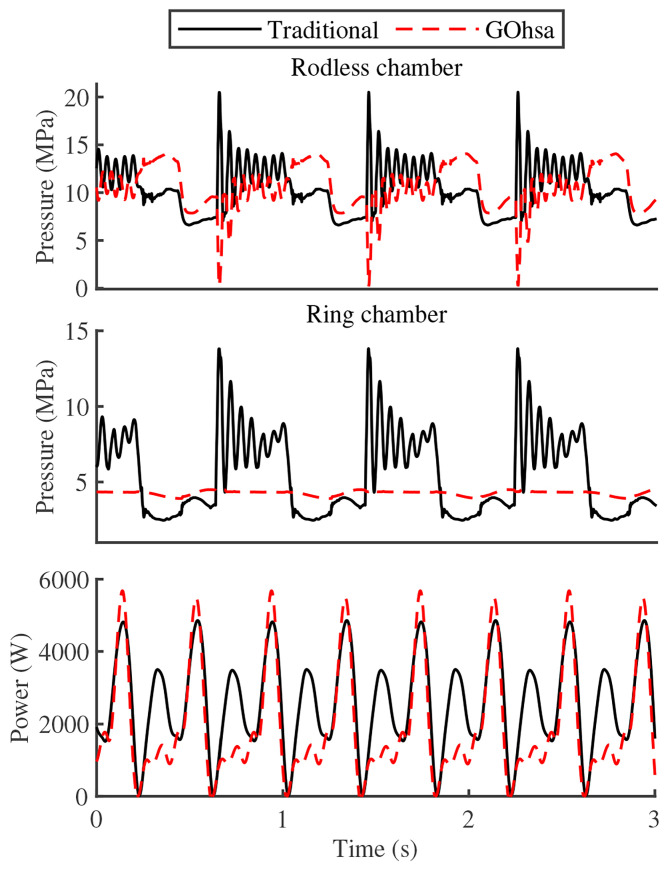
Chamber pressure and power characteristics of the leg actuator.

Figure 7 illustrates the chamber pressure characteristics of the knee-driven unit along with the variable pump’s real-time power consumption profile. The pressure measurements demonstrate that the GOhsa system exhibits lower average pressure values and smaller fluctuation amplitudes, with particularly notable improvement in the pressure characteristics of the rod chamber. This suggests that the GOhsa system can drive the same load with a lower pressure differential, effectively reducing wasted energy consumption and enhancing energy efficiency. The power analysis further verifies the significant reduction in instantaneous power requirement during the swing phase, unequivocally validating the system’s energy-saving advantages.

### 4.2. Experiment and Analysis

#### 4.2.1. Experimental Platform and Setup

**Figure 8:**
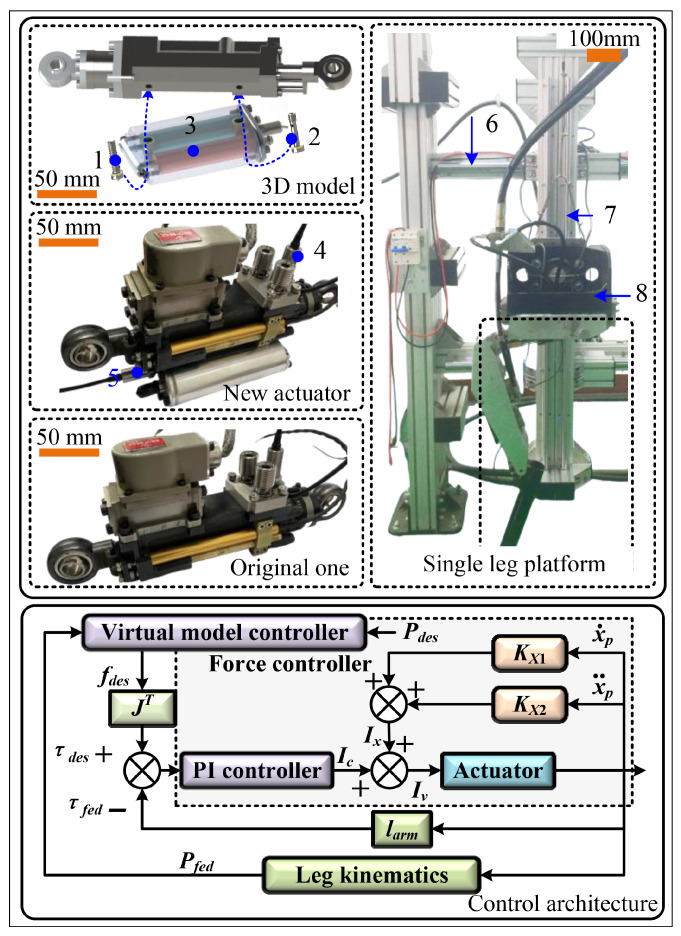
Experimental platform and control architecture.

As illustrated in Figure 8, the experimental frame consisted of a horizontal main beam (labeled 6) and a vertical auxiliary beam (labeled 7), achieving low-damping, low-friction planar motion through linear bearings. The leg assembly comprised a hip fixation frame serving as counterweight (labeled 8), limb linkages, and actuators, with a total weight of approximately 55 kg. The leg was controlled by a Raco9063 controller from National Instruments, abbreviated NI, running on a UNIX-like real-time operating system. Analog signals from position, pressure, and force sensors were acquired using an NI9220 16-bit module at a sampling rate of 1 kHz. Valve control was managed by an NI9264 16-bit output module, linked to a voltage-controlled current source circuit. The EBIUM-00000P pressure sensor measured the oil pressure in the ring chamber, with a measurement range of 10 MPa and accuracy of 0.01 MPa. A fixed indoor hydraulic station powered the single-leg motion, with supply pressure set at 20 MPa.

The GOhsa was derived from a modified existing traditional hydraulic servo actuator. According to the analysis data from previous chapters, the theoretical working pressures in the ring chamber and gas chamber were relatively low. Consequently, the gas chamber and auxiliary oil chamber were fabricated as an independent module (labeled 3) using aluminum alloy 6061, which was then connected to the hydraulic cylinder by two bolts. To ensure communication between the auxiliary oil chamber and the actuator ring chamber, the forward connecting bolt was designed with a central axial bore and multiple radial ports (labeled 1 and labeled 2). Sealing between the independent module and hydraulic cylinder was achieved using O-rings, while brass gasket seals provided sealing at bolt–module interfaces. For real-time working chamber pressure monitoring, miniature hydraulic pressure sensors (labeled 4 and 5) were incorporated on both the independent module end-face sealing cover and the inlet/outlet valve block. Since the sealed cavity formed by the auxiliary oil chamber and ring chamber had to remain gas-free during operation, miniature check valves capable of withstanding vacuum-assisted filling process were installed at the end-face cover. The GOhsa driving control still used a three-position, four-way electro-hydraulic servo valve, with the B port, originally leading to the ring chamber, sealed using a mechanical seal. Key components included: servo valve (HY WISTAR: HY130), hydraulic cylinder (HY WISTAR: HC-197), displacement sensor (VISHAY: REC 38L 03C 502B), force sensor (SRI: M3626AP), and pressure sensor (EBIUM-00000P-100BAR). The structural dimensions are consistent with the data listed in Table 3.

Based on the virtual model control strategy, a motion control framework for the leg assembly was constructed as shown in Figure 8, building upon the force control law discussed in previous chapters. Here, $l_{arm}$ denotes the force arm corresponding to the joint actuator, and $J^{T}$ represents the transpose of the force Jacobian matrix. The variables *P*, *f*, and τ denote the foot-end position, virtual foot-end force, and corresponding joint torque, respectively. The subscripts des and fed denote desired and feedback values.

Before the experiment, the oil injection check valve was connected to a vacuum filling machine for evacuation. Subsequently, high-pressure air was charged into the gas chamber. Hydraulic oil was then injected into the GOhsa ring chamber via the vacuum filling machine. The final stabilized oil pressure was precisely controlled using the pressure sensor to ensure that some oil would remain in the auxiliary oil chamber even after complete piston retraction.

The entire experimental process consisted of two steps:

(1) The first step tested foot-end position response under no-load conditions (simulating the swing phase in actual robot walking). To achieve this, the leg assembly was suspended, and the hip fixture degrees of freedom were locked in both horizontal and vertical directions.
(2) The second step tested foot-end position response under loaded conditions (simulating the stance phase in actual robot walking). In this case, the leg assembly maintained a standing posture, and the hip fixture was allowed to move freely in both horizontal and vertical directions. High-frequency squatting motions were then performed.

During the experiment, the parameters $K_{x2}$ and $K_{x1}$ were set to 0.04 A·s/m and 0, respectively. In the virtual model control strategy, the stiffness and damping coefficients were set to 20,000 N/m and 50 N/(m/s) in the horizontal direction, and 35,000 N/m and 30 N/(m/s) in the vertical direction, respectively.

#### 4.2.2. Experimental Results of the First Step

**Figure 9:**
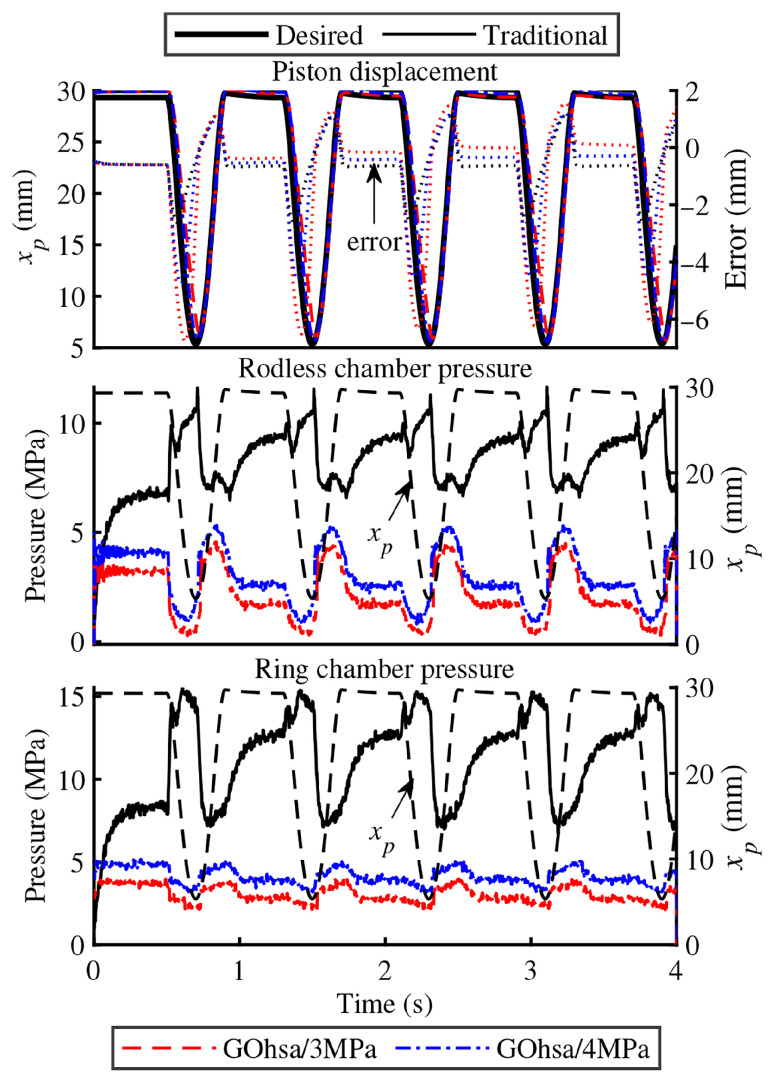
Piston displacement and chamber pressure of the knee-driven unit under no-load tests. The black dashed line represents the displacement of the main piston (*x_p_*), indicated by the arrow in the figure.

Figure 9 illustrates the piston displacement curve and chamber pressure of the knee-driven unit. For performance evaluation, the real-time tracking error curve is superimposed on the displacement response plot to visually demonstrate the system’s tracking performance. The color of each error curve corresponds exactly to that of its original data, ensuring consistent visual association. During the experiment, GOhsa’s initial inflation pressure was set to 3 MPa and 4 MPa, while the foot-end trajectory followed a cubic spline interpolation curve, with step length and step height both set to 0.2 m and a step frequency of 1.25 Hz. For comparison, a traditional joint actuator was also included in the experiment. The experimental results indicated that under no-load conditions, the GOhsa position response speed and accuracy exhibited only minor differences compared to the traditional actuator. Moreover, as initial inflation pressure increased, tracking performance was further improved.

As can be seen from the pressure curves, the pressures in the two working chambers of GOhsa no longer follow the variation pattern observed in conventional asymmetric hydraulic cylinders with symmetric valve control. The pressure fluctuations are smaller, and the fluctuation range in the ring chamber is reduced compared with that in the rodless chamber. The abrupt pressure jumps caused by piston switching are no longer evident. These observations indicate that the actuator control characteristics are theoretically improved.

#### 4.2.3. Experimental Results of the Second Step

**Figure 10:**
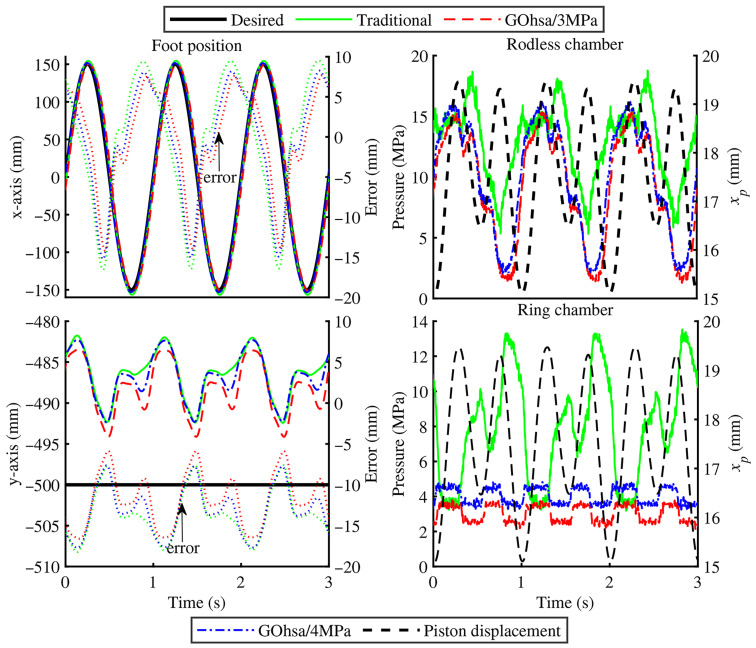
Foot position and chamber pressure of the knee-driven unit under loaded tests.

During the loaded experiment, the foot-end position relative to the thigh pitch joint (reference coordinate point) was [0 m, −0.55 m]. To simulate center of gravity motion during the stance phase of an actual quadruped robot, the foot-end trajectory was planned as a horizontal sinusoidal curve having an amplitude of 0.15 m and a frequency of 1.25 Hz, while maintaining constant vertical position. Figure 10 shows the foot-end position curves along the horizontal (x-axis) and vertical (y-axis) directions based on inverse kinematics as well as the chamber pressure of the knee-driven unit. Due to the absence of gravity compensation in the vertical direction, the leg assembly exhibited certain pre-compression in the standing posture. However, position fluctuation in the vertical direction remained relatively small, staying within approximately 0.01 m. Moreover, in the horizontal direction, the GOhsa actuator continued to demonstrate high position response speed and accuracy. Quantitatively, Figure 10 indicates that the traditional actuator exhibited maximum position tracking errors of 16.6 mm and 18.1 mm along the x- and y-axes, respectively. In contrast, the GOhsa actuator achieved lower errors of 13.7 mm (x-axis) and 16.5 mm (y-axis) under an inflation pressure of 3 MPa, and 15.0 mm (x-axis) and 17.8 mm (y-axis) under 4 MPa. These results clearly indicate that GOhsa maintains equivalent tracking precision to the traditional actuator and demonstrates improved motion stability under load.

Similar to the first step results, the pressures in both GOhsa working chambers continued to fluctuate within a low numerical range. Most notably, when the piston transitioned from extension to retraction, the pressure surge caused by imbalanced inlet/outlet oil flow elevated the pressure in the traditional joint actuator rodless chamber to nearly 20 MPa (the maximum preset system pressure). In contrast, the GOhsa pressure maintained significantly lower levels. This indicates that GOhsa can achieve the same driving performance as traditional actuators using relatively lower supply pressure, representing one of its potential energy-saving advantages.

**Figure 11:**
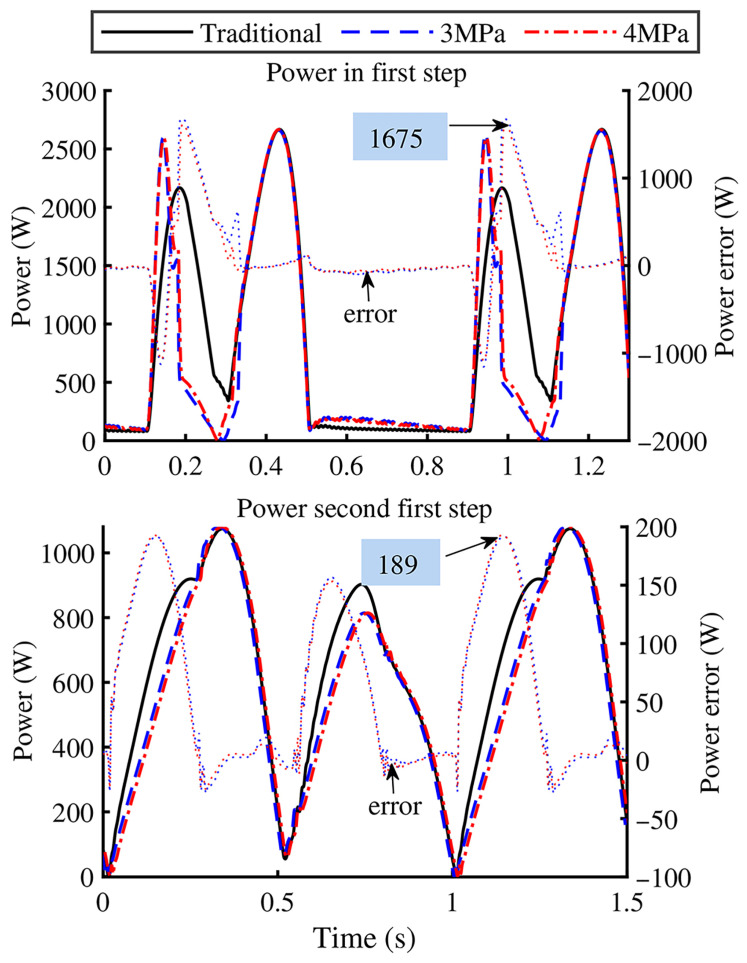
Power curves of the experiments.

Figure 11 shows the leg assembly power curves. Due to the absence of pressure and flow sensors in the experimental system main hydraulic circuit, the pressure values upstream of the valve were based on the theoretical pump setting. Additionally, the system flow rate only considered the actuator operational flow, without accounting for flow losses due to internal leakage and overflow. The actuator power is calculated using the product of pressure and flow rate. The calculation results showed that, compared to traditional actuators, GOhsa power consumption is significantly reduced during piston retraction. Compared to the original structure, under inflation pressures of 3 MPa and 4 MPa, in the first-stage experiment, the maximum power reduction reached 1675 W (79.1%), with average reductions of 156.6 W (15.2%) and 118.2 W (11.5%), respectively. In the second-stage experiment, the maximum power reduction was approximately 189 W (28.0%), with average reductions of 59.9 W (10.0%) and 58.7 W (9.8%), respectively. However, no notable difference in power consumption was observed during the extension phase.

Since GOhsa energy efficiency is primarily attributed to its ability to reduce system flow during piston retraction, its performance exhibits marked sensitivity to foot-end trajectory. This explains why the two experimental groups exhibit markedly different power-saving characteristics specifically during the piston retraction phase.

## 5. Conclusions

To address the low energy efficiency of hydraulically driven quadruped robots, this study proposes a gas–oil hybrid joint actuator based on analysis of quadruped robot topological structure and joint load characteristics. By incorporating a high-pressure gas chamber and auxiliary oil chamber separated by a sliding piston, the ring chamber is passively supplied with low-pressure oil, while pressure in the rodless chamber is actively regulated via an electro-hydraulic servo valve. To develop a force control strategy, a numerical model was established to investigate pressure response characteristics in the ring chamber under friction and damping loads, as well as the relationship between frequency response and initial gas charging pressure. Results indicated that when inflation pressure exceeded 2 MPa, the sliding piston no longer exhibited stick-slip behavior even in the low-speed range. Moreover, at 3 MPa inflation pressure, the unit cutoff bandwidth under loaded conditions reached 2.52 Hz, fully meeting normal quadruped robot actuation requirements. Based on theoretical analysis results, this study developed a force controller for GOhsa using linearization theory and validated its effectiveness and responsiveness through a single-leg experimental platform. Experimental results demonstrated that, compared to conventional hydraulic servo actuators, GOhsa exhibited smaller pressure fluctuation ranges and lower average pressure in its working chamber during operation. This indicates that, under the same compressive load, GOhsa can achieve the same driving capability as traditional units while operating at lower system supply pressure. Furthermore, the energy-saving capacity of GOhsa is primarily reflected in the reduced main oil circuit flow rate during piston retraction, demonstrating that its energy efficiency is strongly dependent on the operating trajectory. This work embodies a principle observed in mammalian leg actuation, where actuation roles depend on gait phase. In air, leg positioning generally requires far less actuation effort than ground contact support and force modulation. GOhsa reflects this role allocation by using passive ring chamber pressurization during the return stroke and by retaining active single-chamber pressure regulation for load-bearing force control. This mapping improves energy efficiency while maintaining bidirectional position and force controllability.

Looking forward, beyond the hydraulic architecture and control framework investigated here, further improvements may come from system level integration. Recent advances in flexible electronics suggest several directions for biomimetic applications. Dynamically morphing microelectronics and micro-origami functional networks may enable ultraflexible, mesh-like modules that conform to joint surfaces [39]. Neuromorphic devices and sensors may support low-power, event-driven processing of high-bandwidth multimodal signals [40]. Active matrix integrated magnetosensitive e-skins and sensor arrays may enable distributed contact and magnetic field sensing for biomimetic perception [41]. Integrating such distributed sensing and embedded intelligence with the hybrid hydraulic actuator could support actuator health monitoring, including diagnosis of sealing, leakage, and precharge state, and could improve contact-aware control at the leg level. These directions will be explored in future work.

## Figures and Tables

**Table 1:** Specifications of the traditional joint-driven unit used by SCalf-II.

Specifications	Symbols	Values
Piston diameter	$d_{pis}$	0.025m
Piston rod diameter	$d_{r}$	0.012m
Effective bulk modulus	$\beta_{e}$	$0.7\times 10^{3}\;\mathrm{MPa}$
Initial pressure of gas chamber	$P_{g0}$	1/2/3MPa
Displacement of the main piston	$x_{p}$	0–0.072 m

**Table 2:** Parameters of the simulation system.

Symbols	Values
*B*	500 N/(m/s)
$F_{C}^{p}$/$F_{C}^{s}$	100 N/80 N
$F_{S}^{p}$/$F_{S}^{s}$	200 N/150 N
${\dot{x}}_{s}$	0.01 m/s
β	1
$x_{v}^{max}/i_{v}^{max}$	0.0688
$\omega_{v}$	80 Hz
$\xi_{v}$	0.8
$m_{p}$/$m_{s}$	0.28 kg/0.1 kg

**Table 3:** Simulation model supplementary parameter settings.

Specification	Symbol	Value
Effective area of Piston I	$A_{g}$	$3.14\times 10^{-4}\;m^{2}$
Piston I stroke	$X_{g}$	0.1m
Piston II stroke	$X_{o}$	0.072m
Rated flow of control valve	$Q_{pis0}$	24L/min
Valve spool response frequency	$\omega_{v}$	20Hz
Supply pressure	$Q_{S}$	20MPa
Return pressure	$Q_{T}$	0MPa

## Data Availability

The original contributions presented in this study are included in the article. Further inquiries can be directed to the corresponding author. All authors have read and agreed to the published version of the manuscript.

## References

[1] Biswal P., Mohanty P.K. (2021). Development of quadruped walking robots: A review. Ain Shams Eng. J..

[2] Taheri H., Mozayani N. (2023). A study on quadruped mobile robots. Mech. Mach. Theory.

[3] Hua Z., Zhang Z., Chai H., Li Y., Li X., Sun Y. (2023). Energy efficiency onboard hydraulic power for quadruped robot based on high-low double pumps supply. J. Mech. Sci. Technol..

[4] Harper M.Y., Nicholson J.V., Collins E.G., Pusey J., Clark J.E. Energy efficient navigation for running legged robots. Proceedings of the 2019 International Conference on Robotics and Automation (ICRA).

[5] Gonzalez C. (2015). What’s the difference between pneumatic, hydraulic, and electrical actuators. Mach. Des..

[6] Hwangbo J., Lee J., Dosovitskiy A., Bellicoso D., Tsounis V., Koltun V., Hutter M. (2019). Learning agile and dynamic motor skills for legged robots. Sci. Robot..

[7] She J., Feng X., Xu B., Chen L., Wang Y., Liu N., Zou W., Ma G., Yu B., Ba K. (2025). Bionic Energy-Efficient Inverse Kinematics Method Based on Neural Networks for the Legs of Hydraulic Legged Robots. Biomimetics.

[8] Guenther F., Vu H.Q., Iida F. (2019). Improving legged robot hopping by using coupling-based series elastic actuation. IEEE/ASME Trans. Mechatron..

[9] Cho B., Kim M.S., Kim S.W., Shin S., Jeong Y., Oh J.H., Park H.W. (2021). Design of a compact embedded hydraulic power unit for bipedal robots. IEEE Robot. Autom. Lett..

[10] Cho B., Kim S.W., Shin S., Kim M.S., Oh J.H., Park H.W. (2021). Energy efficient control of onboard hydraulic power unit for hydraulic bipedal robots. J. Korea Robot. Soc..

[11] Cho B., Kim S.W., Shin S., Oh J.H., Park H.S., Park H.W. (2022). Energy-efficient hydraulic pump control for legged robots using model predictive control. IEEE/ASME Trans. Mechatron..

[12] Dong J., Jin B., Zhai S., Liu Z., Cheng Y. (2021). Planning and analysis of centroid fluctuation gait for hydraulic hexapod robot. IEEE Access.

[13] Zhuang Y., Wang Y., Ding Y. Kinodynamic Model Predictive Control for Energy-Efficient Locomotion of Legged Robots with Parallel Elasticity. Proceedings of the 2025 IEEE International Conference on Robotics and Automation (ICRA).

[14] Tanfener E. (2022). Design and Experimental Verification of a Clutched Parallel Elastic Actuation Mechanism for Legged Locomotion. Master’s Thesis.

[15] Culha U., Saranli U. Quadrupedal bounding with an actuated spinal joint. Proceedings of the 2011 IEEE International Conference on Robotics and Automation.

[16] Chu Z., Luo J., Fu Y. Variable stiffness control and implementation of hydraulic sea based on virtual spring leg. Proceedings of the 2016 IEEE International Conference on Mechatronics and Automation.

[17] Zhou S., Chen G., Gong M., Liu J., Xu P., Liu B., Yin N. (2025). Bio-Inspired Compliant Joints and Economic MPC Co-Design for Energy-Efficient, High-Speed Locomotion in Snake-like Robots. Biomimetics.

[18] Zhao H., Zhou J., Ma S., Du S., Liu H., Han L. (2023). Design and experiments of electro-hydrostatic actuator for wheel-legged robot with fast force control response. Machines.

[19] Du S., Zhou J., Zhao H., Ma S. (2024). Research on the energy transfer and efficiency performance of an electro-hydrostatic actuator for a wheel–legged robot joint. Energy.

[20] Du S., Zhou J., Hong J., Zhao H., Ma S. (2024). Application and progress of high-efficiency electro-hydrostatic actuator technology with energy recovery: A comprehensive review. Energy Convers. Manag..

[21] Fan W., Liu T., Yi J., Huang X., Zhang B., Zhang X., Wang S. A passive hydraulic auxiliary system designed for increasing legged robot payload and efficiency. Proceedings of the 2021 IEEE International Conference on Robotics and Automation (ICRA).

[22] Xue Y., Yang J., Shang J., Wang Z. (2014). Energy efficient fluid power in autonomous legged robotics based on bionic multi-stage energy supply. Adv. Robot..

[23] Xue Y., Yang J., Shang J., Xie H. (2015). Design and optimization of a new kind of hydraulic cylinder for mobile robots. Proc. Inst. Mech. Eng. Part C J. Mech. Eng. Sci..

[24] Guglielmino E., Semini C., Yang Y., Caldwell D., Kogler H., Scheidl R. Energy efficient fluid power in autonomous legged robotics. Proceedings of the Dynamic Systems and Control Conference.

[25] Yang C., Zhou L., Wang J., Xu T., Yang C., Ye G. (2023). Research on energy saving system of hydraulic excavator based on three-chamber accumulator. J. Energy Storage.

[26] Liu Y., Xu Z., Hua L., Zhao X. (2020). Analysis of energy characteristic and working performance of novel controllable hydraulic accumulator with simulation and experimental methods. Energy Convers. Manag..

[27] Yu B., Li H., Gu C., Wang X., Zhang L. (2025). Design of lightweight hydraulic power unit for legged robots based on the Sobol sensitivity analysis. Energy Convers. Manag..

[28] Yu B., Li H., Ma G., Liu X., Chen C., Zheng B., Ba K., Kong X. (2024). Design and matching control strategy of electro-hydraulic load-sensitive hydraulic power unit for legged robots. Energy.

[29] Lorke M., Willen M., Lucas K., Beyerbach M., Wefstaedt P., Murua Escobar H., Nolte I. (2017). Comparative kinematic gait analysis in young and old Beagle dogs. J. Vet. Sci..

[30] Yang G., Yao J. (2020). High-precision motion servo control of double-rod electro-hydraulic actuators with exact tracking performance. ISA Trans..

[31] Armstrong-Helouvry B., Dupont P., Canudas-de-Wit C. (1994). A Survey of Models, Analysis Tools and Compensation Methods for the Control of Machines with Friction. Automatica.

[32] Olsson H., Åström K.J., Canudas-de-Wit C., Gäfvert M., Lischinsky P. (1998). Friction Models and Friction Compensation. Eur. J. Control.

[33] Merritt H.E. (1967). Hydraulic Control Systems.

[34] Ruderman M. Full- and Reduced-Order Model of Hydraulic Cylinder for Motion Control. Proceedings of the 43rd Annual Conference of the IEEE Industrial Electronics Society (IECON 2017).

[35] Boaventura T., Semini C., Buchli J., Frigerio M., Focchi M., Caldwell D.G. Dynamic torque control of a hydraulic quadruped robot. Proceedings of the 2012 IEEE International Conference on Robotics and Automation.

[36] Levine W.S. (2018). The Control Handbook (Three Volume Set).

[37] Han B., Si S., Luo Q., Xiao D., Niu K. (2016). Co-simulation of a quadruped robot’s mechanical and hydraulic systems based on ADAMS and AMESim. J. Beijing Inst. Technol..

[38] Sun Y., Hua Z., Li Y., Chai H., Li X., Su B. (2021). Modeling and analysis on low energy consumption foot trajectory for hydraulic actuated quadruped robot. Int. J. Adv. Robot. Syst..

[39] Merces L., Ferro L.M.M., Thomas A., Karnaushenko D.D., Luo Y., Egunov A.I., Zhang W., Bandari V.K., Lee Y., McCaskill J.S. (2024). Bio-Inspired Dynamically Morphing Microelectronics toward High-Density Energy Applications and Intelligent Biomedical Implants. Adv. Mater..

[40] Merces L., Ferro L.M.M., Nawaz A., Sonar P. (2024). Advanced Neuromorphic Applications Enabled by Synaptic Ion-Gating Vertical Transistors. Adv. Sci..

[41] Becker C., Bao B., Karnaushenko D.D., Bandari V.K., Rivkin B., Li Z., Faghih M., Karnaushenko D., Schmidt O.G. (2022). A New Dimension for Magnetosensitive E-Skins: Active Matrix Integrated Micro-Origami Sensor Arrays. Nat. Commun..

